# Combining Piezoelectric Stimulation and Extracellular Vesicles for Cartilage Regeneration

**DOI:** 10.1155/2023/5539194

**Published:** 2023-06-29

**Authors:** Chengteng Lai, Fei Jin, Zhangqi Feng, Rui Zhang, Meng Yuan, Lili Qian, Lei Zhang, Yongxiang Wang, Jianning Zhao

**Affiliations:** ^1^Nanjing Jinling Hospital, Affiliated Hospital of Medical School, Nanjing University, Nanjing, China; ^2^School of Chemistry and Chemical Engineering, Nanjing University of Science and Technology, Nanjing 210094, China; ^3^Center for Public Health Research, Medical School and Jiangsu Key Laboratory of Molecular Medicine, Nanjing University, Nanjing 210002, China; ^4^Department of Orthopaedics, Northern Jiangsu People's Hospital, The Affiliated Hospital of Nanjing University Medical School, Yangzhou 225001, China

## Abstract

Numerous patients experience articular cartilage defects (ACDs), which are characterized by progressive cartilage degradation and often lead to osteoarthritis (OA). Consequently, 44.7% of OA patients suffer from dyskinesia or disability. Current clinical drug treatments offer limited effectiveness in fully curing the disease. In this study, we propose a collaborative approach that combines physical and biological cues to promote cartilage regeneration. A biodegradable piezoelectric poly (l-lactic acid) (PLLA) nanofiber scaffold facilitates in situ, battery-free electrical stimulation under natural joint loading, while extracellular vesicles (EVs) serve as communication mediators between cells and promote cell proliferation, migration, and secretion of type II collagen. In this combined approach, EVs attached to PLLA are gradually released by localized piezoelectric electrical stimulation and taken up by chondrocytes. This process results in the organization of type II collagen along the PLLA fiber surface, ultimately forming cartilage lacunae that facilitate the residence of new chondrocytes. As an outcome, a significant round cartilage defect (diameter: 3 mm and depth: 1 mm) in the PLLA/EVs group (rat and knee) was rapidly restored within six weeks. In contrast, individual EVs and PLLA groups demonstrated considerably weaker cartilage regeneration capabilities. This research suggests that the synergistic effect of electromechanical stimulation and EVs-based biological cues is a crucial intervention method for treating OA.

## 1. Introduction

Osteoarthritis (OA) is the leading cause of joint pain symptoms and poses a risk of disability for patients [1–3]. Current treatment methods, such as drug therapy, tissue grafts, and synthetic functional scaffolds, demonstrate limited efficacy in cartilage regeneration [4, 5]. Drug therapy can only alleviate inflammation and pain to a certain degree without curing the disease, while tissue grafts are constrained by donor availability and prone to complications such as immune reactions, infection, and donor site morbidity. Synthetic scaffolds, or artificial grafts, have garnered significant interest due to their unrestricted supply and customizability for specific purposes, but they tend to degrade under repeated joint forces. Consequently, there is a need for alternative solutions that promote chondrogenesis and cartilage regeneration. Acknowledging this limitation, we turn to nature for inspiration. Cartilage is known to be sensitive to bioelectric electrical stimulation (ES) [6]. Numerous studies have demonstrated the effectiveness of exogenous ES in promoting cartilage regeneration [7, 8]. However, most of these treatments necessitate external energy input and wired connections, which can lead to risks such as infection, reduced efficacy, and device failure. Recent advances in nanotechnology and smart materials enable in situ ES treatments with a high spatial resolution [9, 10]. Electromechanical coupling piezoelectric materials have been employed in tissue regeneration engineering but have not been widely accepted for electrically stimulating cartilage cells. This is because the implanted piezoelectric scaffold can only generate sufficient stimulating potential under substantial deformation, whereas joint mobility in OA patients is typically limited. Extracellular vesicles (EVs) function as crucial biological cues that can facilitate chondrogenesis [11–13]. Importantly, based on the principle of electroporation [14], EV cargos can be effectively transferred to receptor cells under the continuous local action of ES, as it increases membrane permeability. In this study, we present a collaborative strategy for stimulating hyaline cartilage regeneration using a cell-free piezoelectric poly (l-lactic acid) (PLLA) nanofiber scaffold combined with EVs. Although research on EVs-seeded electrospun scaffolds for cartilage repair is limited, there has been a successful case of skin tissue repair using electrospun polycaprolactone-EVs scaffolds [15]. In our prior research, the long noncoding RNA DANCR was found to play a crucial role in the chondrogenesis of synovium-derived mesenchymal stem cells (SMSCs) [16]. Studies have suggested that the therapeutic potential of MSCs is derived from their ability to secrete EVs [17, 18]. To investigate this, we established a DANCR-overexpressed SMSC cell line (DANCR + SMSCs) and extracted its EVs for examination. Through six weeks of animal clinical trials, the self-powered biodegradable scaffold with DANCR + SMSC-EVs (DS-EVs) displayed high efficiency in promoting rapid cartilage regeneration, eliminating the need for invasive removal procedures typically required for conventional piezoelectric scaffolds such as poly (vinylidene difluoride).

## 2. Materials and Methods

### 2.1. Cell Culture and Transfection

To overexpress lncRNA DANCR and obtain DANCR + SMSCs, the recombinant full-length rat DANCR cDNA was cloned into the pcDNA3.1 vector (Thermo Fisher Scientific, Inc.). The pcDNA3.1 empty vector served as a negative control. SMSCs (60–70% confluence) were transfected with pcDNA3.1-DANCR or pcDNA3.1-NC using Lipofectamine® 2000 (Invitrogen; Thermo Fisher Scientific, Inc.). Briefly, Lipofectamine 2000 was mixed with 50 nM plasmids, added to SMSCs in 10 cm cell culture dishes, and incubated for 6 h at 37°C. Subsequently, cells were cultured in DMEM + 10% FBS for 24 h and used for further experiments. Transfection efficiency was assessed via reverse transcription-quantitative PCR (RT-qPCR). For more details on primary cell isolation and identification, please refer to Support Information S1.

### 2.2. Isolation and Identification of DANCR + SMSC-EVs

Once DANCR + SMSCs reached 90% confluence (10 cm cell culture dishes), the culture medium was replaced with serum-free medium and incubated continuously. The cell supernatant was collected three days later. DS-EVs were isolated by differential centrifugation (Support Information S1) and identified by Western blot for DS-EVs surface markers (Syntenin-1 and CD81); the size and concentration of DS-EVs were determined by Nanoparticle Tracking Analysis (NTA); and the DS-EVs morphology was examined under a transmission electron microscope (TEM). All identification experiments on DS-EVs, including Western blot, NTA, and TEM, were performed in triplicate.

### 2.3. Nanoparticle Tracking Analysis

The number and size distribution of DS-EVs were analyzed by nanoparticle tracking using Electrophoresis and Brownian Motion Laser Scattering Microscopy (ZetaView, Germany). Briefly, samples in solution or each particle sample were diluted in PBS, and their relative concentrations were determined according to the dilution factor. Data analysis was conducted with NTA 2.2 software. For each sample, a set of at least four individual movies was acquired, and the count was averaged.

### 2.4. Transmission Electron Microscopy

DS-EVs were resuspended in 2.5% glutaraldehyde in 0.1 M sodium cacodylate buffer and fixed on formvar carbon-coated grids at room temperature for 30 min, followed by negative staining with 2% uranyl acetate for 1 min. Images were captured using a transmission electron microscope (JEM-2100, JEOL).

### 2.5. Cell Migration and Proliferation Studies

The isolated and identified DS-EVs were resuspended in 50–100 *µ*L PBS, and their concentrations were determined using an Enhanced BCA Protein Assay Kit (P0010, Beyotime, China).

A chondrocyte scratch test was conducted to assess the impact of DS-EVs on isolated chondrocyte migration. Briefly, approximately 1.2 × 10^5^ cells (per well) were initially transferred to a 6-well plate and incubated for 24 h until the cells reached nearly 95% confluence. The prepared DS-EVs/PBS solution, diluted separately to 0 *µ*g/mL, 20 *µ*g/mL, 40 *µ*g/mL, and 60 *µ*g/mL concentrations, was added to the six-well plates and continuously observed for 0 h, 6 h, 12 h, and 24 h under a light microscope. Images were recorded with a digital microscope to examine the migration capabilities of chondrocytes.

To assess chondrocyte proliferation, an MTT assay was performed to study the effect of DS-EVs on chondrocyte proliferation. Isolated rat chondrocytes (0.5 × 10^4^ cells/well) were transferred to 96-well plates and treated with sodium nitroprusside, followed by a 60 *µ*g/(mL *∗* day) treatment of DS-EVs for 24, 72, and 120 h, after which proliferation was evaluated. MTT reagent (100 *µ*L, 10%) was added to each well for 4 h, and the wells were analyzed using a microplate reader, with optical activity observed at 570 nm.

### 2.6. Western Blot Assays

Isolated DS-EVs pellets and chondrocytes co-cultured with DS-EVs for varying durations were extracted with RIPA buffer (Santa Cruz) containing a protease inhibitor cocktail (Med ChemExpress) and incubated on ice for 30 min. Protein content of DS-EVs and chondrocyte lysates was quantified using a BCA kit (Thermo Fisher Scientific), loaded onto SDS-PAGE (10% gels), and transferred to PVDF membranes (Millipore). The membranes were blocked for 1 h in 3% BSA and incubated with corresponding antibodies, including Syntenin-1 and CD81, overnight at 4°C. Secondary antibodies were IRDye 680RD antirabbit immunoglobulin (Ig)G (H + L) and IRDye 800CW antimouse IgG (H + L) (Invitrogen). Detection was performed with an Odyssey Imaging System (LI-COR, Biosciences).

### 2.7. Quantitative Real-Time PCR

Cells and ground animal tissue were washed with PBS twice before RNA purification. RNA was purified using TRIZOL following the manufacturer's protocol. The concentration and purity were determined using a NanoDrop 2000 spectrophotometer (Thermo Fisher). cDNA was synthesized using an RT2 First Strand Kit (Qiagen). Quantitative reverse transcription polymerase chain reaction (qRT-PCR) was conducted using GoTaq qPCR Master Mix (Promega) on an ABI 7900 (Applied Bioscience). Relative gene expression was calculated using the 2^−ΔΔ^CT method, with glyceraldehyde-3-phosphate dehydrogenase (GAPDH) serving as an internal control (see Table 1).

### 2.8. Fabrication and Characterizations of PLLA/DS-EVs Scaffold

PLLA nanofibers (NFs) were fabricated through electrospinning using a #21 nozzle. For additional information on the electrospinning process, please refer to Supporting Information S2. Subsequently, the as-received PLLA NFs mat was immersed in the DS-EVs suspension. The morphology and structural distribution were examined using SEM (Supra 55, Carl Zeiss) and TEM (JEM-2100, JEOL). Piezoelectric response measurements of individual NFs were carried out using PFM (Multimode 8, Bruker) in contact mode. For further information on PFM characterizations, consult Supporting Information S3.

### 2.9. Detection of the DS-EVs Retention Rate of PLLA/DS-EVs

Straightforward modifications to the electrospinning conditions can produce PLLA fibers with minimal piezoelectric effects. In this study, 4000-rpm PLLA nanofiber was used to fabricate piezoelectric PLLA, while 300-rpm PLLA served as a nonpiezoelectric control.

Both types of prepared PLLA/DS-EVs (thickness: 1 mm; volume: 7.07 mm^3^; loaded with 300 *µ*g DS-EVs) were placed in a sterile cell culture dish and soaked with 1 mL sterile PBS. Then, 1 mL of PBS was collected every 24 h. The material's surface was repeatedly washed and squeezed 10 times in 1 mL of freshly added PBS, and the sterile PBS was continuously collected for 10 days. An NTA particle size analyzer was employed to measure the DS-EVs concentration in the sterile PBS collected daily and to calculate the adsorption rate, adsorption amount per unit volume, and retention rate of PLLA on DS-EVs.

Preprepared DS-EVs stained with DiR dye were used to assess the in vivo EVs retention rate. Two randomly selected SD rats were used, with one receiving a DS-EVs^DiR^ injection and the other receiving a PLLA/DS-EVs^DiR^ implant. The fluorescence of DS-EVs was analyzed for two weeks using an LB983 in vivo imaging system (Berthold Technologies).

### 2.10. Animal Models

Fifty-seven adult male SPF-grade SD rats, weighing between 280 and 310 g, were utilized in this study. Three rats were employed for primary chondrocyte extraction and other in vivo experiments. The remaining rats were randomly assigned to the MOCK group (blank control), Sham group (Sham surgery), ACD group (defect diameter: 3 mm; depth: 1 mm), PLLA implanted group, DS-EVs injection group, and PLLA/DS-EVs implanted group. For further information on the animal models, please refer to Support Informations S4 and S5.

The rats were provided by Shanghai SIPPR-BK Laboratory Animal Co. Ltd., and the quality of the experimental rats was verified by the Shanghai Laboratory Animal Quality Supervision and Inspection Station. The use and breeding of experimental animals were approved by the Department of Comparative Medicine, Jinling Hospital, Nanjing University School of Medicine (Approval No. 2020JLHKJLWDWLS-001).

### 2.11. Pathological Staining and Immunohistochemistry

Femur histological sections were decalcified with EDTA, fixed with paraformaldehyde, and embedded in paraffin. Rat femur sections were then cut into 4 *µ*m slices. Hematoxylin-Eosin (H&E) staining, toluidine blue staining, and safranin O staining were performed on bone sections following the manufacturer's instructions. In addition, COL2 antibody (Proteintech 1 : 200) and MMP13 antibody (Abcam 1 : 200) were diluted according to the recommended ratios, and immunohistochemistry was conducted on femoral sections.

### 2.12. Biomechanics Analysis

The experiment was divided into three groups: Sham (*n* = 3), control (*n* = 3), and PDS-EVs treatment (*n* = 3). Rat knee joint specimens were prepared as flat surfaces using a wire saw. An MML Nano Indenter was used to perform biomechanics analysis for each group. The maximum loading force was set at 20 mN, with the loading position situated at the deep layer of cartilage and a loading time of 15 s. A cylindrical indenter from Hysitron, USA, with a bottom diameter of 10 *µ*m, was selected for this experiment.

### 2.13. Microcomputed Tomography (Micro-CT)

The distal femur of each rat was isolated, fixed in 4% paraformaldehyde, and scanned using a Bruker Micro-CT SkyScan 1276 system (Kontich, Belgium). Scan settings included: voxel size 6.533712 *µ*m1, medium resolution, 85 kV, 200 *µ*A, 1 mm Al filter, and an integration time of 384 ms. Density measurements were calibrated to the manufacturer's calcium hydroxyapatite (CaHA) phantom. The analysis was conducted using the manufacturer's evaluation software. Reconstruction was performed by NRecon (version 1.7.4.2). 3D images were obtained from contoured 2D images using methods based on distance transformation of the grayscale original images (CTvox; version 3.3.0). 3D and 2D analyses were carried out with CT Analyzer software (version 1.18.8.0).

### 2.14. Statistical Analysis

The software GraphPad 8.0.1 was utilized for graphical illustration and statistical analysis. The outcomes are presented as means ± standard deviation (SD) derived from three autonomous experiments unless indicated otherwise. The divergence within treatment groups was assessed through the Student's *t*-test or one-way analysis of variance (ANOVA) along with Bonferroni's multiple comparisons test.

## 3. Results

### 3.1. Construction of Bionic Scaffold and Its Mechanism in Cartilage Regeneration

**Figure 1:**
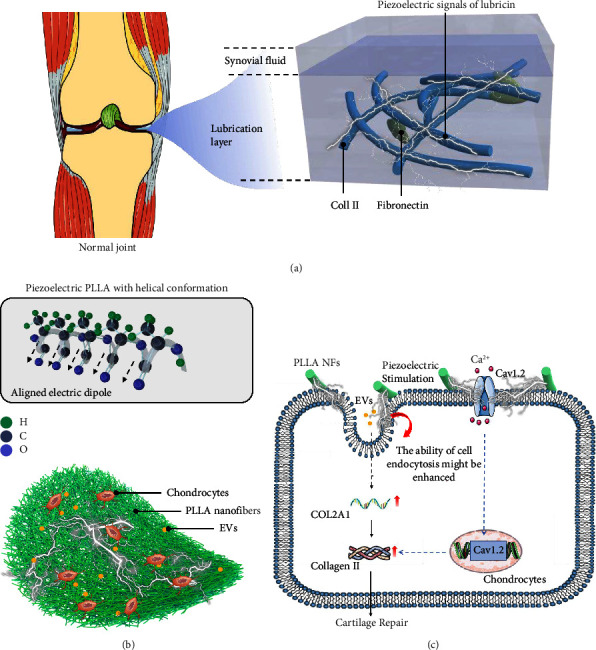
Construction of bionic scaffold and its mechanism in cartilage regeneration: (a) microstructure of normal articular cartilage; (b) chemical structure of PLLA and schematic diagram of the construction of PLLA/EVs composite scaffold; (c) schematic diagram of the mechanism of PLLA/DS-EVs involved in repairing articular cartilage defects.

The physiological structure of the distal femur knee joint can be divided into the cartilage layer, calcified layer, and subchondral bone layer. The cartilage layer is characterized by collagen fibers that form grid-like cartilage lacunae. Within these lacunae, articular chondrocytes can reside and secrete glycosaminoglycans and other essential components of the cartilage matrix [19, 20]. Research has demonstrated that periodic mechanical stimulation can activate the calcium ion channel Cav1.2 in chondrocytes and enhance the expression of type II collagen [21, 22]. The PLLA scaffold utilized in this study exhibited positive piezoelectricity and could convert mechanical energy into bioelectrical energy (Support Information, S2B–S2D) [23, 24]. The mammalian knee cartilage experiences body weight and mechanical compression during static or dynamic processes. Therefore, the PLLA scaffold is a suitable option for cartilage tissue engineering (Figures 1(a) and 1(b)).

Extracellular vesicles contain genetic information and perform most of the functions of the originating cell. These vesicles primarily facilitate cell communication through paracrine, proximal secretion, and endocrine mechanisms. This phenomenon is frequently observed in MSCs [17, 18, 25–29]. Our previous research demonstrated that the lncRNA DANCR can regulate chondrogenic differentiation and proliferation [16]. Furthermore, it was found that DANCR may have a role in inducing chondrogenesis [30]. In addition, in this study, we made an initial discovery that DANCR may have the capacity to upregulate the synthesis of COL2A1. Subsequently, we generated DANCR + SMSCs and isolated their extracellular vesicles for further investigation. We hypothesized that DS-EVs could elicit comparable or analogous biological effects to those of DANCR + SMSCs.

In this study, we proposed a therapeutic approach for repairing ACD that involved combining PLLA with DS-EVs to form EV-seeded electrospun scaffolds. The PLLA NFs could serve as a bridge to promote the migration of normal chondrocytes toward the center of cartilage defects. Furthermore, during the process of cell migration, the piezoelectric stimulation produced by daily knee joint activities may promote chondrocyte uptake of DS-EVs, which are slowly released from PLLA/DS-EVs [31]. Ultimately, this uptake of DS-EVs may enhance the ability of chondrocytes to synthesize type II collagen, thereby promoting cartilage repair (Figure 1(c)) (speculation). Notably, PLLA fiber scaffolds possess a grid-like structure similar to collagen II, making it a suitable biomimetic material. Moreover, PLLA does not produce harmful substances during the degradation process and exhibits excellent tissue compatibility [32, 33].

### 3.2. Extraction of DS-EVs and Validation of Its Effect on Cartilage Regeneration

Traditional tissue engineering technology involves seed cells, scaffolds, cytokines, and the microenvironment. However, maintaining the viability of seed cells in vitro poses a significant challenge, hindering its clinical applications. As a result, research on decellularized scaffolds has garnered significant attention among scientists [34, 35].

**Figure 2:**
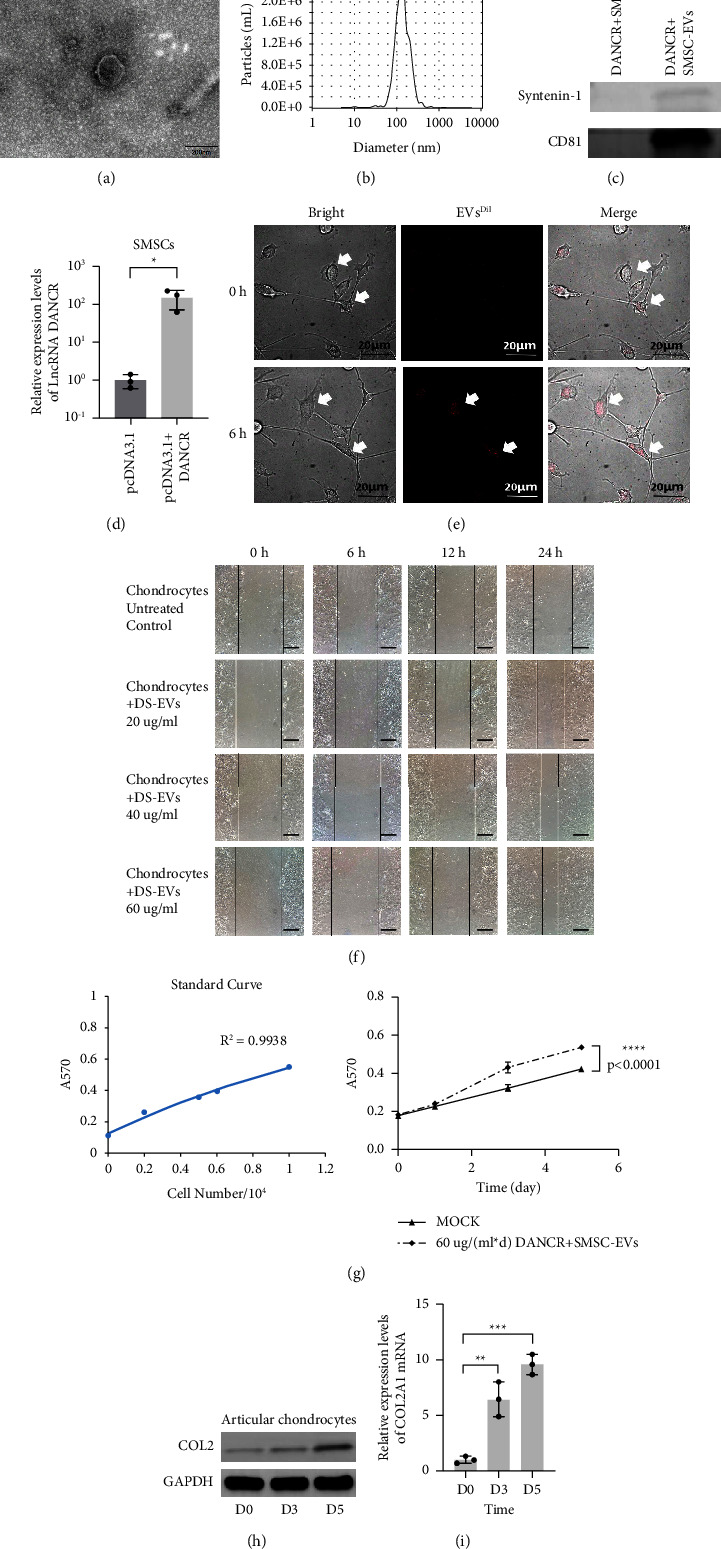
Extraction of DS-EVs and validation of its effect on cartilage regeneration (a) classical spherical structure with phospholipid molecular layer observed by DS-EVs in TEM; (b) NTA particle size analysis: the mean diameter of DS-EVs is 130.6 nm; (c) detection of DANCR + SMSC-EVs surface antigens by Western blotting: CD81 and Syntenin-1 positive; (d) detection of DANCR overexpression plasmid transfection efficiency of SMSCs by qPCR: DANCR + SMSCs were successfully constructed, and their DANCR expression level was 273-fold higher than the MOCK group (^*∗*^,*p*=0.0372); (e) extracellular vesicles fluorescence tracing experiment: the fluorescently labeled DS-EVs entered the chondrocyte matrix after co-culture with articular chondrocytes for 6 h (bar = 20 *µ*m); (f) cell scratch test: After chondrocytes were treated with 60 *µ*g/mL sterile DS-EVs suspension for 24 h, the cell migration ability was significantly enhanced (bar = 500 *µ*m); (g) MTT experiment: chondrocytes were treated with 60 *µ*g/mL sterile DS-EVs suspension for five consecutive days, and the cells were found to proliferate profoundly; (h) Western blotting results: chondrocytes were continuously treated with 60 *µ*g/mL sterile DS-EVs suspension for 3 and 5 days, and the secretion of type II collagen increased; (i) RT-qPCR results: chondrocytes were continuously treated with 60 *µ*g/mL sterile DS-EVs suspension for 3 and 5 days, and the expression levels of COL2A1 increased(^*∗∗*^, *p*=0.004; ^*∗∗∗*^, *p*=0.0001).

**Figure 3:**
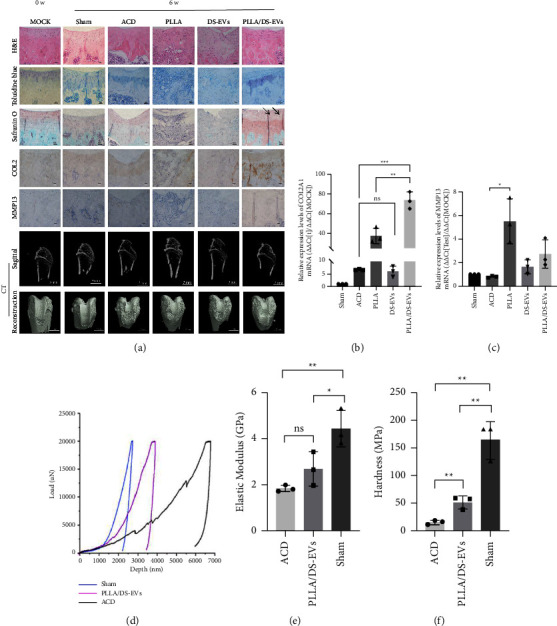
Synergistic effect between piezoelectric stimulation and EVs together enhances the regeneration of cartilage: (a) pathological staining (bar = 50 *µ*m), immunohistochemistry (bar = 50 *µ*m; black arrows: the tidal line), and CT scan (bar = 3 mm) results in different groups; (b) relative expression levels of COL2A1 mRNA in different groups (^*∗∗*^, *p*=0.0058; ^*∗∗∗*^, *p*=0.0002); (c) relative expression levels of MMP13 mRNA in different groups (^*∗*^, *p*=0.0132); (d) nanoindentation experiments: load-depth curves; (e) nanoindentation experiment: Young's modulus of the ACD group, PLLA/DS-EVs group, and Sham group (^*∗*^, *p*=0.0494; ^*∗∗*^, *p*=0.005); (f) nanoindentation experiment: hardness of ACD group, the PLLA/DS-EVs group, and the Sham group (ACD group vs. PLLA/DS-EVs group:^*∗∗*^, *p*=0.0073; ACD group vs. Sham group: ^*∗∗*^, *p*=0.0014; PLLA/DS-EVs group vs. Sham group: ^*∗∗*^, *p*=0.0049).

This study aimed to investigate the biological function of DS-EVs. Our transmission electron microscopy results demonstrated that the isolated DS-EVs had a classical phospholipid layer spherical structure with a diameter of approximately 120–130 nm (Figure 2(a); Supporting Information, S6). NTA analysis further confirmed that the diameter of DS-EVs was approximately 130.6 nm (Figure 2(b); Supporting Information, S6). Western blot analysis revealed that the isolated DS-EVs expressed CD81 and Syntenin-1 (Figure 2(c)). To verify the successful construction of SMSCs overexpressing DANCR, we performed qPCR (Figure 2(d)). Subsequently, we labeled the isolated DS-EVs with Dil dye and co-cultured them with articular chondrocytes. Under a laser confocal microscope, we observed that the DS-EVs could penetrate the articular chondrocytes and aggregate in the cellular matrix after 6 h (Figure 2(e)). The outcomes of the cell scratch assay and MTT assay indicated that DS-EVs (60 *µ*g/mL) could enhance the migration and proliferation of chondrocytes (Figures 2(f) and 2(g)). Furthermore, continuous treatment of articular chondrocytes with 60 *µ*g/mL DS-EVs for five days resulted in increased secretion of collagen type II by the cells (Figures 3(h) and 3(i)).

These experimental findings not only suggest that DS-EVs have the potential to repair cartilage but also provide a theoretical basis for utilizing extracellular vesicles tissue engineering technology to address the innate shortcomings of traditional tissue engineering techniques.

### 3.3. Synergistic Effects between Piezoelectric Stimulation and EVs Together Enhances the Regeneration of Cartilage

**Figure 4:**
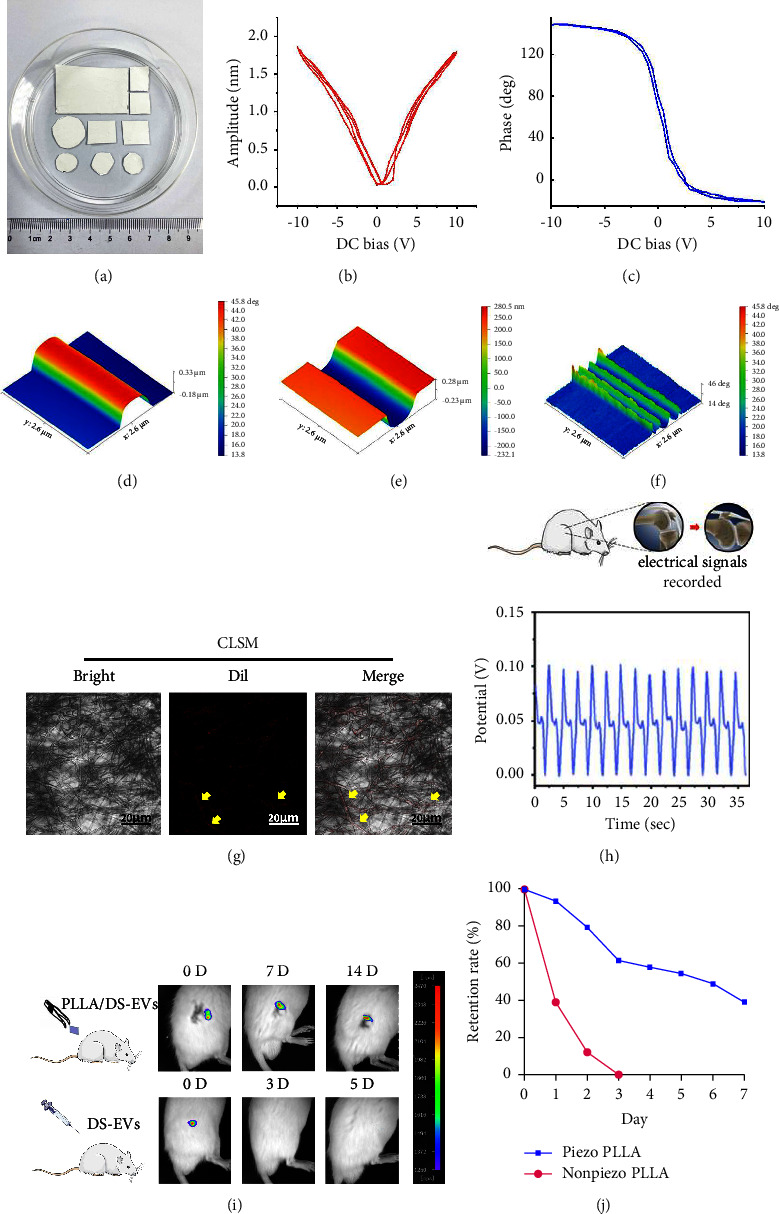
PLLA, PLLA/DS-EVs, and the retention rate of DS-EVs (a) electrospin PLLA nanofibers could be tailored to fit shapes and sizes for later use in vitro and vivo; (b) piezoelectric amplitude plots of the electrospun PLLA nanofibrous membrane; (c) piezoelectric phase plots of the electrospun PLLA nanofibrous membrane; (d) PFM height image; (e) the PFM amplitude image; (f) the PFM phase image; (g) visualization of PLLA/DS-EVs under confocal laser scanning microscope (CLSM): in bright field, PLLA NFs were fibrillary distributed (bar = 20 *µ*m); DS-EVs labeled with DiI dye showed red signals at excitation wavelengths of 549–565 nm (yellow arrows; bar = 20 *µ*m); it could be seen in the merge image that DS-EVs were distributed along the surface of the fibers (yellow arrows; bar = 20 *µ*m); (h) the generated physiological electrical signals were tested during the knee joint activity in rats; (i) in vivo retention of DS-EVs by PLLA NFs; (j) in vitro retention of DS-EVs by PLLA NFs.

Electrospinning was used to prepare the PLLA NFs scaffolds (Figure 4(a)), and subsequently, PLLA/DS-EVs were prepared using a specific process (Supporting Information, S2). The piezoelectric activity of individual PLLA NFs was investigated by piezoresponse force microscopy (PFM), a well-established method for analyzing the local piezoelectric properties of nanostructures [36] (Figures 4(b)–4(f)). The PFM amplitude (Figure 4(b)) and PFM phase (Figure 4(c)) showed a classical butterfly-shaped curve, indicating the hysteresis loops of the PLLA membrane and confirming the good piezoelectric properties of electrospun PLLA NFs, which is a typical feature of piezoelectric materials [37]. Confocal laser scanning microscope images showed the distribution of DS-EVs on the surface of PLLA NFs through electrostatic adsorption (Figure 4(g)). Furthermore, the physiological voltage generated by rats during the knee joint (Figure 4(h)). The movement was measured and found to be within the range of voltage changes produced by PLLA NFs (Supporting Information, S2). To assess the in vivo retention rate of PLLA/DS-EVs, we implanted them into rat knee joints and tracked DiR-fluorescently labeled DS-EVs using small animal imaging technology. The implanted group showed detectable fluorescent signals on day 14, whereas the injected group's signal disappeared by day 3 (Figure 4(i)). This finding indicated that DS-EVs adsorbed on PLLA NFs were slowly released in the in vivo environment compared to direct injection. Next, we analyzed the DS-EVs released from nonpiezoelectric and piezoelectric PLLA collected in vitro for 10 consecutive days using an NTA particle size analyzer to obtain the in vitro retention rate of DS-EVs. The piezoelectric PLLA had a better retention rate of DS-EVs than nonpiezoelectric PLLA, with 40% of total DS-EVs still retained after 7 days (Figure 4(j)).

According to the grading method of the International Cartilage Repair Society (ICRS), injuries with a diameter greater than 3 mm are categorized as articular cartilage defects (ACD). Most of these injuries cannot be fully repaired and are instead filled with fibrocartilage. When the diameter of the ACD exceeds 6 mm, it not only becomes irreparable but also results in further damage to the surrounding bone wall and articular cartilage. This creates a larger defect cavity, which in turn causes the surrounding cartilage to shift and the articular cartilage to collapse. Ultimately, this leads to knee osteoarthritis [38]. By preventing the onset of osteoarthritis during the ACD stage, the incidence of OA can be greatly reduced. Therefore, we chose ACD models (diameter: 3 mm, depth: 1 mm) for this study.

The results of HE staining, toluidine blue staining, and safranin O fast green staining indicated that the PLLA/DS-EVs group exhibited a substantial treatment effect. The HE results revealed that the PLLA scaffolds were not completely degraded in the PLLA/DS-EVs group, which aligns with the reported degradation rate of PLLA [39]. In the PLLA/DS-EVs group, a distinct tidal line was formed, and there were more newly formed chondrocytes residing in the cartilage lacunae. The outcomes of immunohistochemistry demonstrated a higher amount of newly formed COL2A1 in the PLLA/DS-EVs group, while the expression of MMP13 was significantly upregulated in the PLLA group, forming macrophages. The results of immunohistochemistry were consistent with the qPCR outcomes. In addition, based on the CT scanning and 3D reconstruction results, the PLLA/DS-EVs group exhibited a significant repair effect (Figure 3(a); Supporting Information, S7). To understand the changes in the cartilage microenvironment during the repair of articular cartilage, we analyzed the regenerated cartilage tissue around the defects using qPCR and discovered a substantial change in the COL2A1 and MMP13 indexes (Figures 3(b) and 3(c)).

We conducted nanoindentation experiments on various rat knee joint specimens, and the application of PLLA/DS-EVs resulted in favorable biomechanical properties. Specifically, among the groups, only the ACD, Sham, and PLLA/DS-EVs groups had relatively complete cartilage layers after six weeks of ACD model construction, so only these groups were examined (Figure 3(d)). Our results indicated that the PLLA/DS-EVs group exhibited significantly better elastic modulus and hardness compared to the ACD group (Figures 3(e) and 3(f)).

However, we observed that the treatment effect of PLLA alone was not as effective as anticipated. We further validated the expression levels of MMP13 and COL2A1 via qRT-PCR and immunohistochemistry, which demonstrated that the degradation rate of collagen II was higher than its synthesis rate during the repair of ACD using PLLA scaffolds alone. This underscores the importance of the biological function of DANCR + SMSC-EVs in the repair process. It is noteworthy that we did not delve into the specific mechanism of DS-EVs in this study. Based on our prior studies and some of our experimental results, we suggest that DS-EVs carry specific LncRNA from SMSCs, which can regulate cell proliferation and stimulate collagen II production. However, this conclusion requires further experimental evidence in the future.

## 4. Conclusion

Overall, our findings suggest that the combination of PLLA/DS-EVs could enhance the migration and proliferation of normal chondrocytes towards the injury site in the ACD and modulate the expression of COL2A1 and MMP13. Moreover, the impact of piezoelectric stimulation facilitated by the PLLA/DS-EVs scaffold could potentially improve the efficiency of cargo delivery from DS-EVs to regenerated chondrocytes. Eventually, regenerated chondrocytes could secrete type II collagen, which aligns along the PLLA surface, generating cartilage lacunae that facilitate chondrocyte adhesion.

## Figures and Tables

**Table 1:** The following primers were used for qPCR.

Gene	Forward sequence (5′-3′)	Reverse sequence (5′-3′)
rat DANCR	CTCGGATAGAAGCGCAGGTT	AGGCAAGCGGGGTCATTAAA
rat COL2A1	GAGGGCAACAGCAGGTTCAC	TGTGATCGGTACTCGATGATGG
rat MMP13	AACCAGATGTGGAGTGCCTGATG	CACATCAGACCAGACCTTGAAGGC
rat GAPDH	TTGTGCAGTGCCAGCCTC	GGTAACCAGGCGTCCGATAC

## Data Availability

The data that support the findings of this study are available from the corresponding author upon reasonable request.

## Supplementary Materials

Supplementary Materials Supplementary data for this article can be found in the Supplementary Materials file. Supporting information, S1: cell extraction, identification, and isolation of extracellular vesicles; supporting information, S2: preparation of PLLA and PLLA/DS-EVs; supporting information, S3: piezoresponse force microscopy (PFM); supporting information, S4: in vivo micro-MRI; supporting information, S5: details of animal models; supporting information, S6: additional TEM and NTA analysis of DANCR + SMSC-EVs; supporting information, S7: the nonpiezoelectric PLLA was conducted in animal experiments as a negative control.
