# Supplementary material for: Combining Piezoelectric Stimulation and Extracellular Vesicles for Cartilage Regeneration
Source: J Tissue Eng Regen Med. 2023 Jun 29;2023:5539194. doi: 10.1155/2023/5539194 (PMC11919029; doi:10.1155/2023/5539194)
Supplement: Supplementary Materials — Supplementary data for this article can be found in the Supplementary Materials file. Supporting information, S1: cell extraction, identification, and isolation of extracellular vesicles; supporting information, S2: preparation of PLLA and PLLA/DS-EVs; supporting information, S3: piezoresponse force microscopy (PFM); supporting information, S4: in vivo micro-MRI; supporting information, S5: details of animal models; supporting information, S6: additional TEM and NTA analysis of DANCR + SMSC-EVs; supporting information, S7: the nonpiezoelectric PLLA was conducted in animal experiments as a negative control. [file 5539194.f1.pdf]

Supporting Information

Combining Piezoelectric Stimulation and Extracellular Vesicles for Cartilage Regeneration

Running Title: PLLA- EVs scaffold for cartilage regeneration

Chengteng Lai [1#], Fei Jin[2#], Zhangqi Feng[2#], Rui Zhang[4], Meng Yuan[4], Lili Qian[2], Lei Zhang[1*], Yongxiang Wang[3*], Jianning Zhao[1*]

[1]Nanjing Jinling Hospital, Affiliated Hospital of Medical School, Nanjing University, Nanjing, China

[2]School of Chemistry and Chemical Engineering, Nanjing University of Science and Technology, Nanjing 210094, China

[3]Department of Orthopaedics, Northern Jiangsu People's Hospital, The Affiliated Hospital of Nanjing University Medical School, Yangzhou 225001, China

[4]Center for Public Health Research, Medical School and Jiangsu Key Laboratory of Molecular Medicine, Nanjing University, Nanjing 210002, China

#These authors contributed equally to this work.

* Corresponding author

Lei Zhang

E-mail: leizhang1987md@163.com

Yongxiang Wang

E-mail: wangyongxiang@nju.edu.cn

Jianning Zhao

E-mail: zhaojianning.0207@163.com

Section 1. Cell extraction, identification and isolation of extracellular vesicles

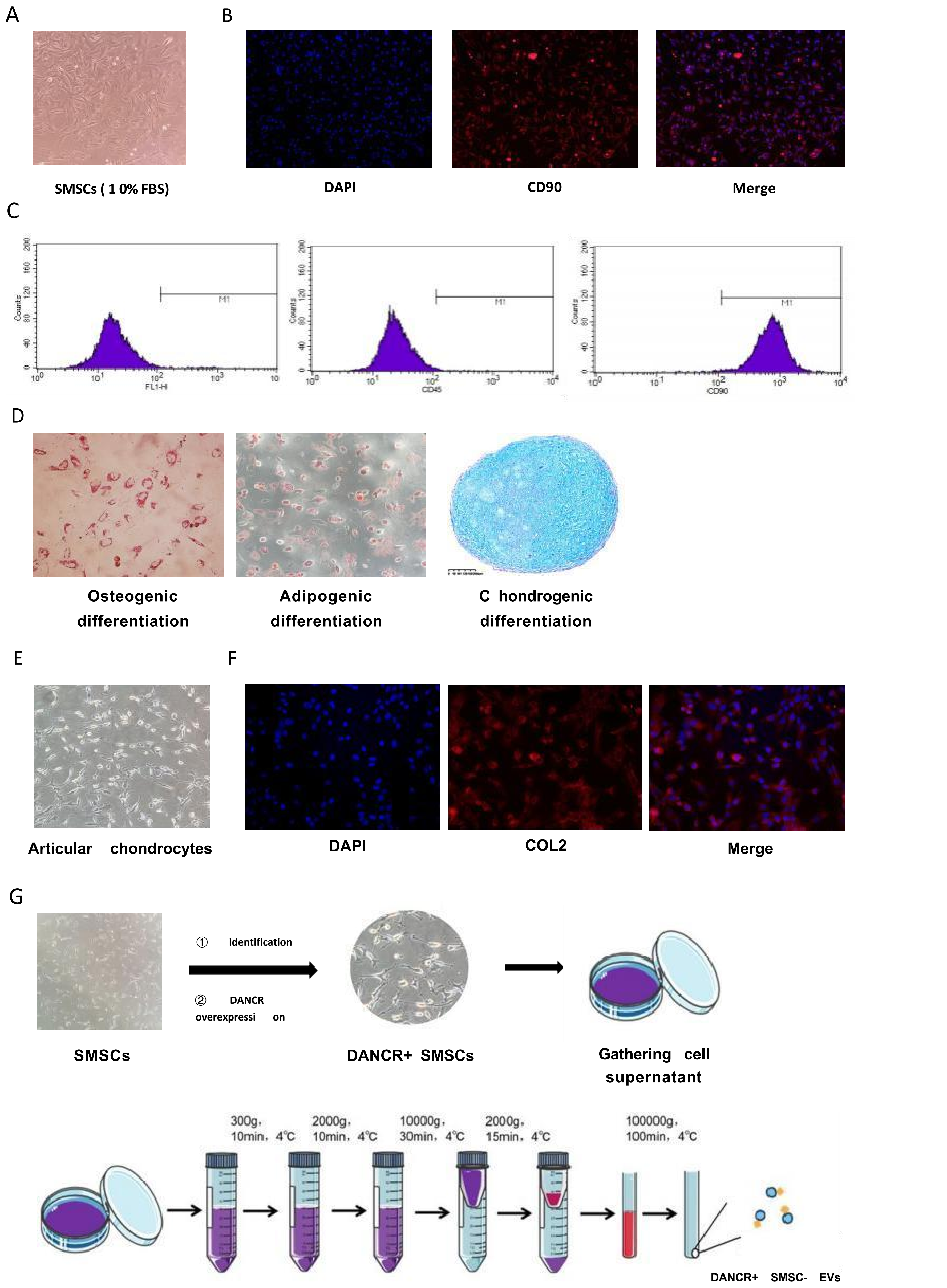

Section 1 . Cell extraction, identification and isolation of extracellular vesicles (A) An SD rat was randomly selected, sacrificed and soaked in 7 5 % alcohol for 5 minutes. The synovial tissue in the joint cavity was taken out, and the articular cartilage and synovial membrane were cut out into 1 mm3 pieces, and submerged in PBS containing benzyl penicillin and streptomycin. The tissue was digested with type II collagenase in 3 7 ° C water bath shaker until no large pieces of tissue remain. After the tissue suspension was filtered through a cell mesh and centrifuged at 1 0 0 0 rpm× 3 min, the supernatant was discarded. The cells were suspended in DMEM medium containing 1 0 % FBS, and cultured in poly- lysine pre- coated culture dishes in a constant temperature incubator at 3 7 ℃ , 5 % CO2 , with first medium change at day 3 . The medium was changed every 3 days afterwards until the cell culture reached full confluence. **(B, C)** The surface antigens of SMSCs were identified by immunofluorescence technology and flow cytometry. **(D)** Culturing SMSCs according to special medium conditions makes SMSCs differentiate into osteoblasts, adipocytes and chondrocytes. **(E)** An SD rat was randomly selected, sacrificed and soaked in 7 5 % alcohol for 5 minutes. The bilateral hips and knee joints cartilage were aseptically cut out on an ultra- clean workbench; the rat articular chondrocytes were extracted using the method described before. **(F)** The surface antigens of articular chondrocytes were identified by immunofluorescence technology. **(G)** The DANCR+ SMSC- EVs were isolated by differential centrifugation .

Section2. Preparation of PLLA and PLLA/DS-EVs

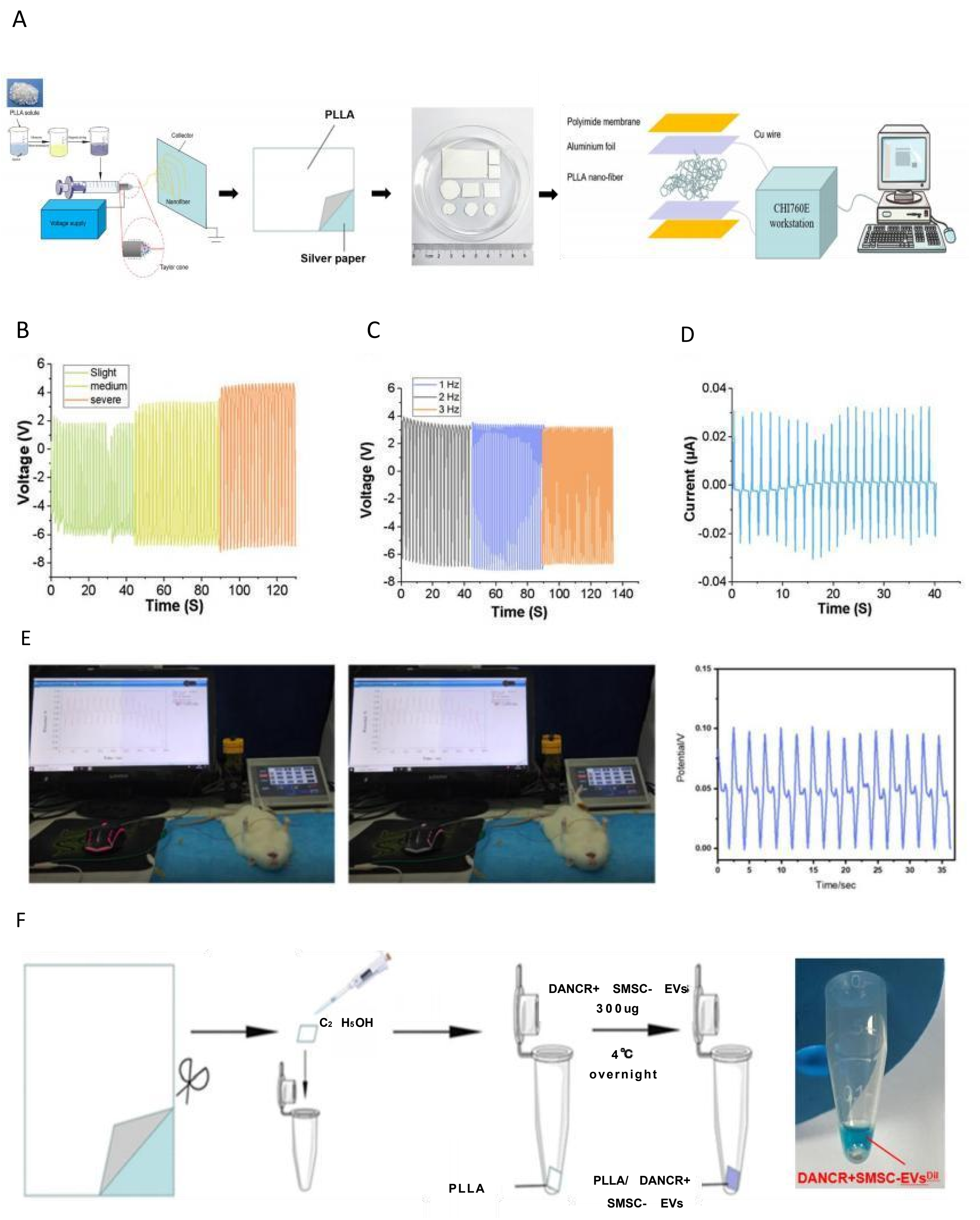

Section2 . Preparation of PLLA and PLLA/ DS- EVs (A) 0.048 g PLLA was placed in a 4 ml glass bottle with a lid and 1.2 ml of dichloromethane was added and stirred to dissolve. Then, 0.3 ml N, N- dimethylformamide was added and stirred continuously for 30 minutes. An 1 ml syringe was used to draw part of the above- prepared PLLA solution and place it on the micro syringe pump with flow rate set at 0.75 ml/ h. The syringe pump would provide the driving force for the movement of the syringe, and the high- voltage power supply would provide for the electric field required for electrospinning. The positive electrode was connected to the middle and lower end of the metal needle tube of the syringe, and the negative electrode was connected to the directional receiving device (using a 20 μ m thick aluminum foil paper plane) , and the applied voltage is about 8.7 kV. The distance between the needle tip and the directional receiver was 10 cm, with ambient temperature of 20 ° C and the humidity at 45 % - 65 % . After peeling off the PLLA membrane prepared above from the aluminum foil, a PLLA fiber membrane was obtained. **(B, C, D)** To test the piezoelectric output performance of the PLLA film, it was necessary to prepare piezoelectric devices based on the nanofibers prepared above. The PLLA nanofibers electrospun on aluminum foil paper were cut into a 1 × 1 cm rectangle, and tightly adhered to another aluminum foil paper of the same size with a conductive adhesive so that the nanofiber membrane was sandwiched between the two sheets of aluminum foil paper. Two copper wires from both sides of the aluminum foil paper were led as output wires. In the end, the entire piezoelectric sensor was packaged with polyimide. The prepared piezoelectric sensor was connected to the CHI760E electrochemical workstation (Shanghai Chenhua Co. , Ltd.) . After the circuit was stabilized, the PLLA was squeezed back and forth through the displacement deformer (extrusion force is 2 N/ cm2) . The output voltage and current of the circuit were measured, and the result was displayed by the CHI760E software. **(E)** Considering that the ACD model was constructed on rats, we needed to know the physiological electrical signal of knee joint. The voltage sensor was attached to one rat' s leg, and the linear motor was used to pull the leg to simulate the electrical signal generated by the movement of the knee joint. **(F)** The PLLA fiber was cut into a circular shape of about 1 cm in diameter which was placed at the bottom of a 1.5 ml test tube. 75 % ethanol was used to drop on the PLLA at the bottom of the tube so that it sticked to the bottom of the tube without suspending. The test tube was dried in a 37 ℃ shaker for 30 minutes. DS- EVs suspension (300 ug/ 50 ul) was added to the tube , stored at 4 ℃ overnight to obtain PLLA/ DS- EVs (PDS- EVs) .

Section 3. Piezoresponse force microscopy (PFM)

The specific measurement method was described as followed: We analyzed the vector (including OP and IP) piezoresponse images and local hysteresis loops in PLLA nanofibers using the dual AC resonance tracking (DART) mode of PFM (Cypher S, Asylum Research). In this study, a train of DC voltage pulses with a constant duration of 25 ms was applied to the tip. The amplitude of the pulses varied stepwise from −10 to 0 V and then from 0 to 10 V and back to −10 V. The nanodomain switching properties of the nanofibers were then resolved in real space with a nanometric resolution by SS-PFM. Au/Cr coated tips were used during the measurement (spring constant = 0.11N m−1, free air resonance frequency = 35 kHz). An AC excitation (Vac= 1.6 V) riding on the DC bias voltage (Vdc) was applied between the tip and the bottom electrode.

Section 4. In vivo Micro-MRI

In vivo 3-T MRI was performed using a 3-T MR scanner (Ingenia, Philips Health Care, Best, the Netherlands), using a 32-channel phased-array head coil. Diagnostic imaging included 3D fluid attenuated inversion recovery (FLAIR), 3D T1-weighted imaging (WI), diffusion WI and susceptibility WI (SWI). 3D FLAIR parameters included a 250 mm field of view (FOV) with a 256 × 184 matrix, a section thickness of 1 mm with a 0.57 mm overlap, no parallel imaging, a repetition time (TR)/echo time (TE) ratio of 6000/378 (shortest), and an inversion time (TI) of 2000 ms; the number of signals acquired was two and the acquisition time was 4 min 42 s. SWI parameters included a FOV of 230 mm with a 320 × 251 matrix, a section thickness of 0.8 mm over contiguous slices, a minIP of 5 mm, a TR/TE ratio of 22/11.5 (in-phase), 37 (shifted); number of signals acquired, 1; flip angle 20。; and an acquisition time of 6 min 42 s. 3D T1WI parameters included a FOV of 260 mm; matrix, 288 × 288; section thickness, 0.9 mm; TR (ms)/TE (ms) ratio, 7.6 (shortest); TE (shortest), 3.6 ms; flip angle, 10。; and an acquisition time of 4 min 42 s.

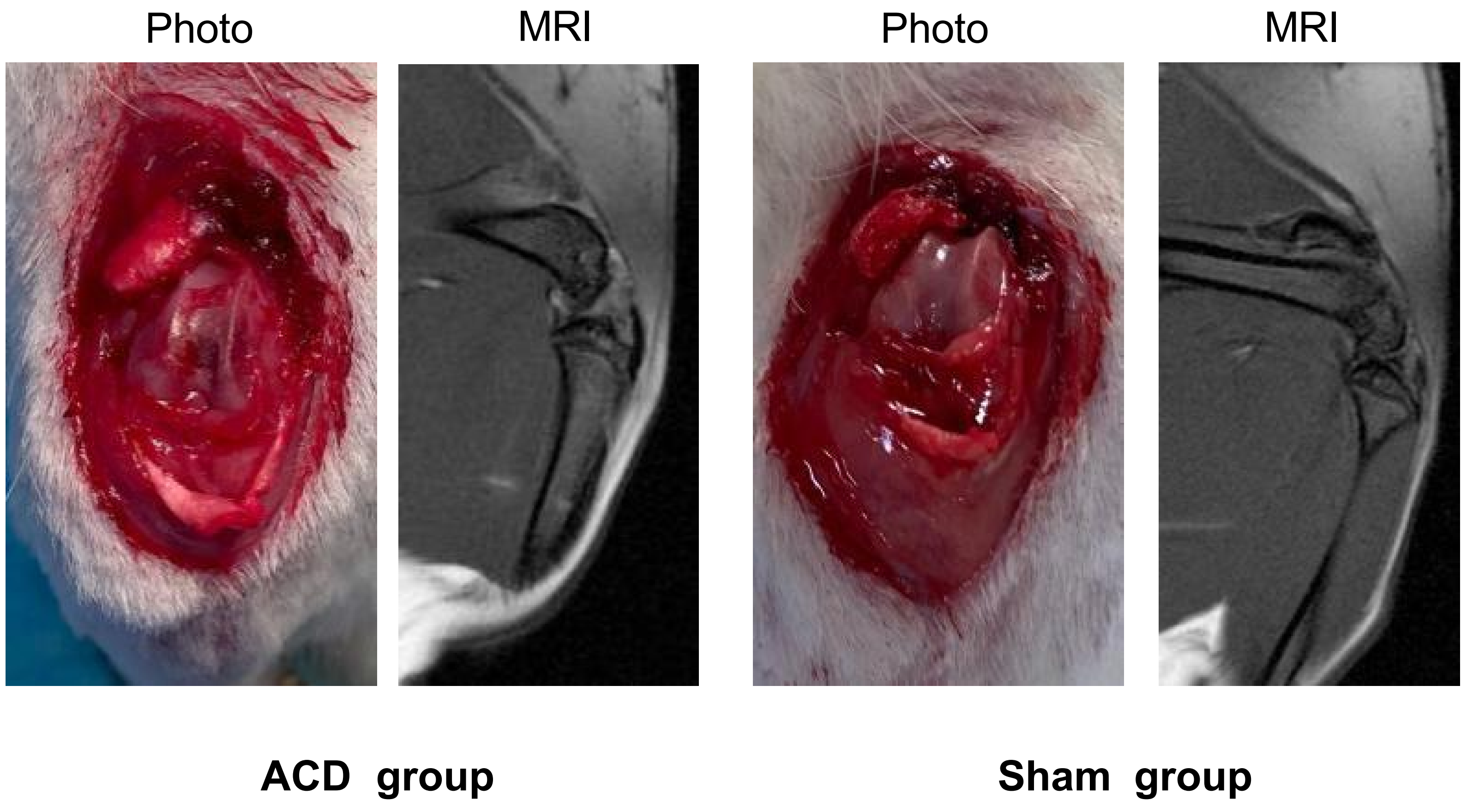

Section 4. Micro-MRI The intraoperative photographs & the MRI images on the first postoperative day were shown.

Section5. Animal models

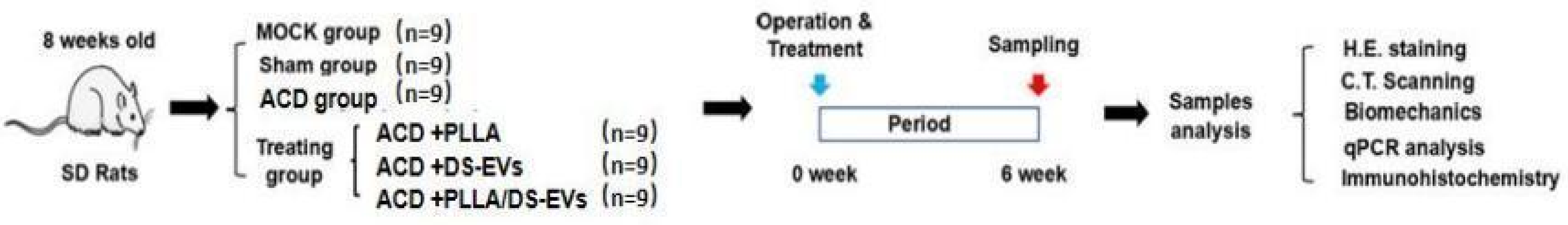

Section 5． Animal models Fifty- four adult male rats were randomly and equally divided into 6 groups, and the rats in each group were sacrificed （ 6 weeks after the operation ） to obtain materials for experimental analysis.

Section6. Additional TEM & NTA analysis of DANCR+SMSC-EVs

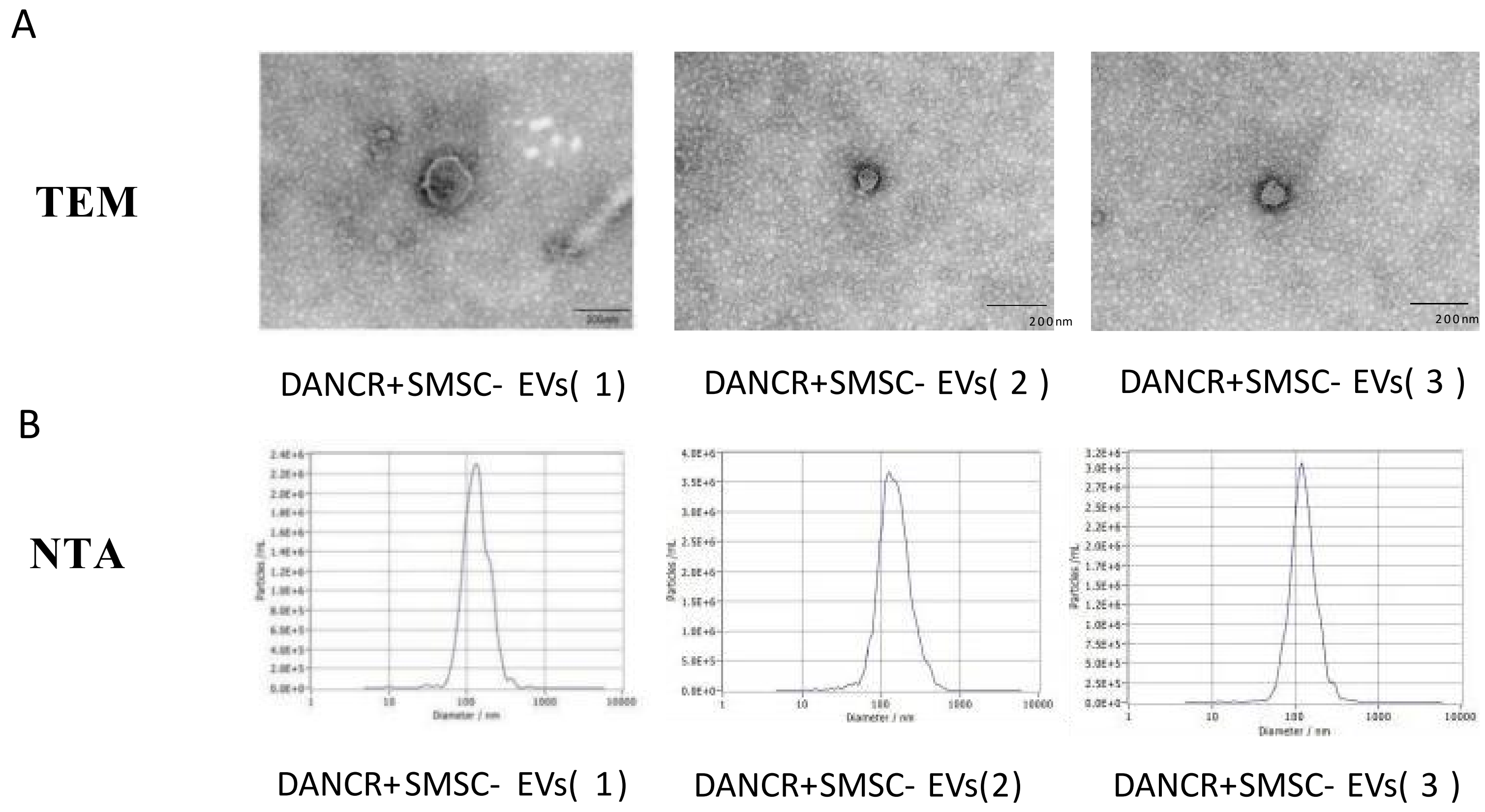

Section 6． Additional TEM & NTA analysis of DANCR+ SMSC- EVs (A) Additional TEM images of DANCR+ SMSC- EVs; (B) NTA particle size analysis: the mean diameter of DANCR+ SMSC- EVs were **1 3 0 . 6 nm; 1 2 7 . 7 nm; 1 2 4 .1nm**

Section7. Supplementary experiment: Nonpiezo PLLA (NC group)

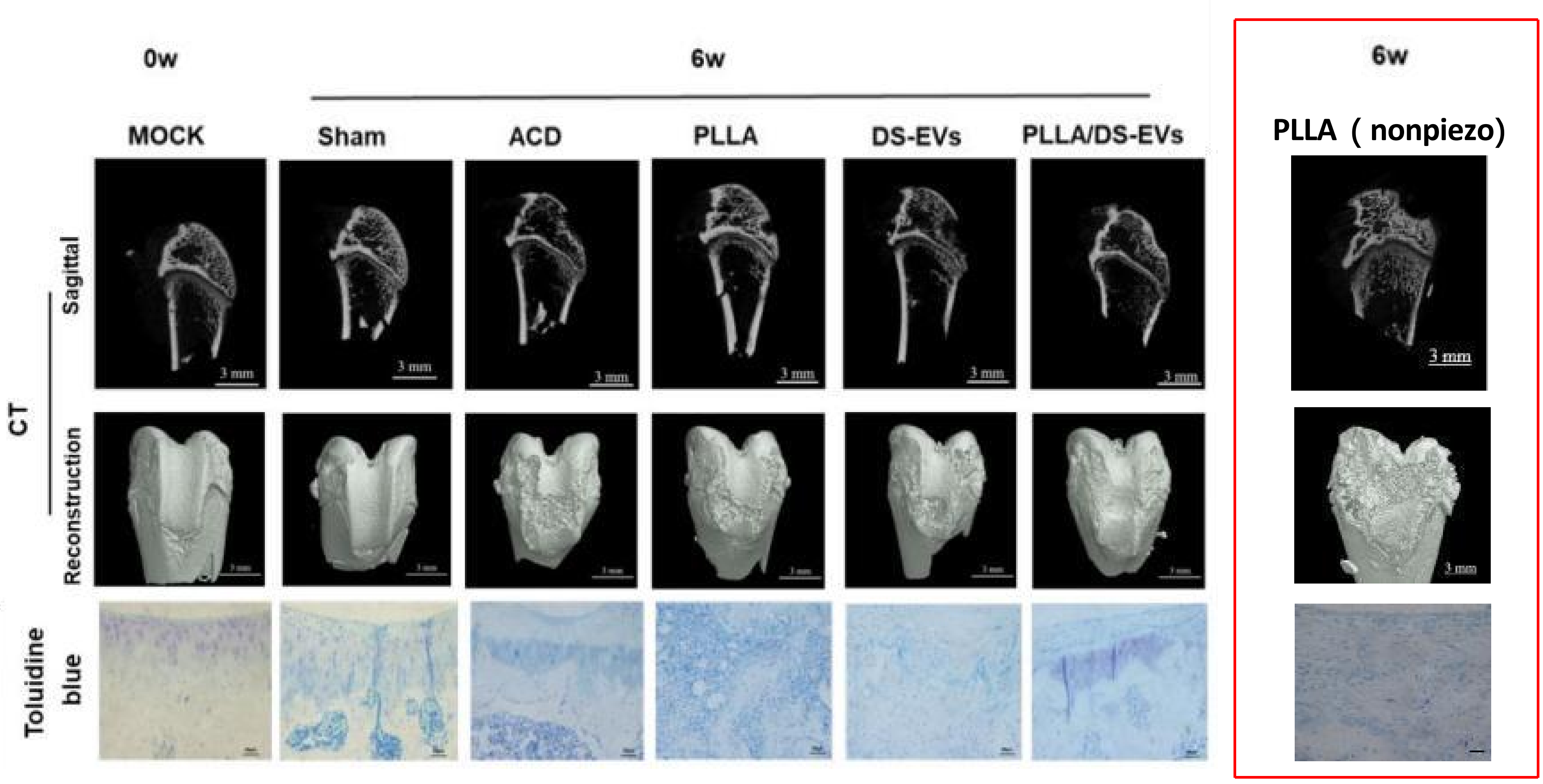

Section 7． Supplementary experiment ： Nonpiezo PLLA (NC group) Toluidine blue staining （bar= 5 0 μ m）， and CT scan （bar= 3 mm） results in different groups
